# Discovery of a specialist Copelatinae fauna on Madagascar: highly ephemeral tropical forest floor depressions as an overlooked habitat for diving beetles (Coleoptera, Dytiscidae)

**DOI:** 10.3897/zookeys.871.36337

**Published:** 2019-08-12

**Authors:** Tolotra Ranarilalatiana, Johannes Bergsten

**Affiliations:** 1 Department of Entomology, Faculty of Sciences, Box 906, Antananarivo University, 101 Antananarivo, Madagascar Antananarivo University Antananarivo Madagascar; 2 Department of Zoology, Swedish Museum of Natural History, Box 50007, SE-10405 Stockholm, Sweden Swedish Museum of Natural History Stockholm Sweden

**Keywords:** Analalava, Betampona, *
Copelatus
*, humid forest, *
Madaglymbus
*, Marojejy, Masoala, new species, Nosy Mangabe, overlooked habitat, protected area, rainy season, semi-terrestrial

## Abstract

Diving beetles are generally aquatic and live submerged in water during larval and adult stages. A few groups have colonised hygropetric habitats and fewer species still can possibly be referred to as terrestrial. Here we describe six new Copelatine species that were mainly found in dry shallow forest floor depressions in the eastern and northeastern lowland humid forests of Madagascar. Three new species are described in each of the two genera *Copelatus* and *Madaglymbus*: *Copelatus amphibius***sp. nov.**, *Copelatus betampona***sp. nov.**, *Copelatus zanatanensis***sp. nov.**, *Madaglymbus kelimaso***sp. nov.**, *Madaglymbus menalamba***sp. nov.**, and *Madaglymbus semifactus***sp. nov.** Diagnosis, description, known distribution, ecology, and conservation notes are provided for each species. All species are illustrated with a dorsal habitus image, ventral and lateral views of the male penis, and parameres. Photographs of the unusual terrestrial habitats where the species were found are provided. *Madaglymbus menalamba***sp. nov.** is also documented with macrophotos and videorecordings of the terrestrial locomotion and behaviour in the field. Although these species should not be classified as terrestrial, or even semi-terrestrial Dytiscidae, they seem to be specialists of very ephemeral aquatic habitats and stay put instead of disperse when the habitat dries up. It is hypothesised that this lifestyle and behaviour on Madagascar is restricted to the high-precipitation humid forest regions mainly in the east. It may also represent a transition step, or stepping-stone, towards becoming fully terrestrial, a step that the few known terrestrial Dytiscid taxa once passed through. It is very likely that this type of habitat is overlooked for aquatic beetles, not only in Madagascar, and the six species herein described may be just the “tip of the iceberg”.

## Introduction

Diving beetles are typically 0.8–48 mm long streamlined aquatic beetles with advanced synchronous hind-leg stroke swimming. They are typical of a variety of water bodies ranging from large rivers to small streams, ponds, marshes, mires, bogs and lakes, forest pools, rock pools, ditches, and canals (Gioria 2014; Jäch and Balke 2008; Balke and Hendrich 2016; Miller and Bergsten 2016). A few groups live in hygropetric, also known as madiculous, habitats. These are seepages, wet rocks along streams or splash zones at the sides of waterfalls forming a millimetre-thin film of water over bedrock (Balke et al. 1997; Gioria 2014; Miller and Bergsten 2016). A number of species have adapted to this environment, and concomitantly locomotion has turned more to crawling, creeping, burrowing or jumping than swimming (Balke and Hendrich 2016).

Even fewer diving beetle species have only been found by sifting litter from terrestrial habitats. Their morphology is notably characterised by the absence, or strong reduction, of natatorial setae on legs and they are therefore tentatively referred to as terrestrial (Brancucci 1979, 1985; Watts 1982; Balke and Hendrich 1996; Brancucci and Hendrich 2010; Toussaint et al. 2016; Miller and Bergsten 2016). The life cycle is not known for any of these, for instance if the adults spend their entire life in terrestrial habitats or if the larval development also takes place out of water. Larvae have never been found for any of the suspected terrestrial species.

While adult dytiscids are typically aquatic, all, as far as known, leave water for pupation and many also leave water for dispersal flights (Miller and Bergsten 2016; Bilton 2014). But diving beetles may also be found in temporarily terrestrial habitats when water has recently dried up. As long as there is still moist or damp habitat it is usually possible to find dytiscids if rock or litter are lifted in recently dried up water pools in for instance riverbeds or pond beds. In our previous experience these have been typical stream, or pond inhabitants, some individuals of which have still to search new water or perhaps take a chance that water will soon return. Such species have always been found in larger quantities in water at other or nearby aquatic sites and have not seemed to be specialists of such habitats or behaviour.

However, in our most recent experience of searching dry forest floor depressions in lowland humid forests of northeastern Madagascar, we came across a handful of Copelatinae species, all undescribed, exclusively or almost exclusively found in such habitats. These seem to be specialist of very ephemeral aquatic habitats as we did not find them in nearby streams or other more permanent water bodies. How often and for how long these depressions fill with water is unknown but there was no water in most of them when visited during the rainy season (February–March 2018). Some where not even depressions but small flat pans along paths that would arguably have just a few millimetres of aquatic habitat and only while raining. In terms of the classification of water beetles into six categories by Jäch (1998), it is unclear if they would pass the definition of “true water beetles” based on “true water beetles are submerged … for most of their adult stage”. The frequency and length of dry and submerged periods of these forest floor depressions and flat pans would need to be followed over the season as well as the natural history and phenology of the beetles. In any case we refrain from labelling these as semi-terrestrial, semi-aquatic, amphibious or amphibiotic following the advice of Jäch and Balke (2008) as these terms are variously defined, overlapping in definition or not. What is most significant is that we seem to have come across a previously largely unknown specialist community of diving beetles in these habitats, at a well-known biodiversity hotspot.

Madagascar is one of the world’s most important biodiversity hotspots (Myers et al. 2000) with an extraordinary level of endemism (Goodman and Benstead 2005). The bulk of this unique biodiversity are forest-dwellers and the richest forests are the eastern humid forests. The humid forests climb the north-south running eastern escarpments from sea-level to montane cloud forests where altitude approaches alpine levels above 2000 m. The biodiversity is often altitudinally structured with a different set of species in lowlands, at midaltitude and at high altitude. Deforestation levels on Madagascar have been devastating, with dire consequences also for freshwater fauna as IUCN redlisting status bears witness off (Máiz-Tomé et al. 2018). Although there is still a fair amount of midaltitude humid forests remaining, very little remain of lowland humid forests, and the largest intact lowland forest is that of the Masoala Peninsula in the northeast. Our fieldwork was conducted here as well as in three additional lowland humid forests.

We describe six new species below and note that forest floor depressions and flat pans in tropical humid regions could be an overlooked habitat for specialist diving beetle communities. We provide photos of habitus and of male genitalia, as well as distribution maps for each species. The unusual terrestrial localities are richly illustrated with photos. For one species we also provide *in situ* photos and video recordings from the type locality documenting terrestrial locomotion. The six species belong to the two genera of Copelatinae known from Madagascar, *Copelatus* Erichson, 1832 and *Madaglymbus* Shaverdo & Balke, 2008. *Copelatus* is a megadiverse genus with worldwide distribution while *Madaglymbus* is endemic to Madagascar and the Comoros. The Madagascar fauna of both genera are in different stages of being taxonomically treated (see under Results).

## Materials and methods

### Fieldwork

New collecting efforts of Dytiscidae were conducted in the four protected areas Masoala NP, Marojejy NP, Betampona RNI and Analalava reserve in 2017 and 2018. In Masoala NP both the south side of the main peninsula with Andranobe as base was visited, and the separate island in the Antongil bay, Nosy Mangabe reserve. Marojejy National Park is located a bit further north and is unique in harbouring continuous forests from lowland to alpine levels. A rather isolated patch of remaining lowland humid forest is that of Betampona 35 km NW of Toamasina. It constitutes a strict nature reserve only accessible for researchers. Analalava reserve finally is a very small remaining humid littoral forest on the lowland east coast about 30 km NE of Betampona.

We targeted shallow forest floor depressions or flat pans that bore signs of occasionally having water by being more moist or with a more clayish soil than sourrounding forest floor. Several sites were directly on paths in the forest. None, except one site in Marojejy NP and one in Masoala NP had any connection or was in proximity of running water. Specimens were sampled with sieves, white pans and by hand searching through the clay, soil and leaf litter. Material was collected into plastic tubes with 95% ethanol for conservation.

Each locality was given a collecting event code and associated metadata included geographic name(s), forest type, locality type, habitat description, eventual disturbance, collecting date and collectors. Altitude, latitude and longitude were recorded with a handheld GPS (Garmin). Each locality was also documented with photographs using a compact Panasonic digital camera.

### Preparations and illustrations

Specimens were examined under dissection microscopes from Leica (M165C and MZ12.5). Genitalia were extracted with a fine forceps or pin from the tip of the abdomen and glued on cards on the same pin as the specimen. Photos of habitus were taken with a Canon EOS 5D Mark II DSLR camera equipped with a MP-E 65 mm 1-5X super macrolens and mounted on a motorised rail (Stackshot) from Cognisys. The system was operated using Canon EOS Utility and Zerene Stacker (Zerene Systems) softwares, the latter also used for stacking the Z-stack of captured images with the PMax or DMap algorithm. Photos of genitalia were taken with a Canon EOS 7D DSLR camera mounted on a BALPRO 1 Universal bellow from Novoflex with a long working distance 10X Plano apochromatic microscope objective from Mitutoyo. The system was mounted on a motorised rail (Stackshot) from Cognisys and operated with the same softwares given above. Photo and video recording of one species in the field was done with a Panasonic Lumix DMC-TZ100 compact camera on a gorillapod.

Label data are given as written and separated by “//” if on separate labels and “|” if on different rows on the same label. All examined specimens (individual mounted specimens, or single alcohol tubes with multiple specimens) have been given unique catalogue numbers and these are listed first, starting with “NHRS” followed by a number made up by four letters and nine digits. A series of specimens with consecutive catalogue numbers are given as a range. Other abbreviations used: GP (Genital Preparation) = male genitalia have been examined, ex. = exemplars (number of individuals), Alc. = in alcohol tube. Coordinates are given for the type localities in decimal degrees format within square brackets, followed by administrative region and district (see Fig. 4D for regions).

### Material and depositories

All specimens examined in this study are registered in the Swedish Museum of Natural History, Stockholm, Sweden (NHRS) collection objects database. They are deposited in the following collections and referred to by the abbreviations (paratype series will be shared with other institutions as well):

**NHRS**Swedish Museum of Natural History, Stockholm, Sweden.

**PBZT/MBC**Parc Botanique et Zoologique de Tsimbazaza/Madagascar Biodiversity Center, Antananarivo, Madagasca.

**DEUA**Department of Entomology, Antananarivo University, Antananarivo, Madagascar.

## Taxonomy

### Copelatus

Taxon classificationAnimaliaColeopteraDytiscidae

Erichson, 1832

a3845fcd-e750-5867-ae07-172d4ca2ee61

#### Remark.

Twenty-five species of *Copelatus* are currently known from Madagascar. All species except those in the *erichsonii* group were recently revised by Ranarilalatiana et al. (in press). The three species described below all belong to the *erichsonii* species group with ten discal and one submarginal elytral striae. Type materials of all *erichsonii* group species described from Madagascar have been examined in the ongoing second part of the revision.

### Copelatus amphibius sp. nov.

Taxon classificationAnimaliaColeopteraDytiscidae

8dd58697-a6f1-5d9e-a211-ed0f0f19fbb4

http://zoobank.org/440DF910-AAFD-4430-BDF8-EE5E59B1907C

Figs 1A , 2A

#### Type locality.

Masoala National Park [15.6713S; 49.9672E] (Madagascar, Analanjirofo region, Maroantsetra)

#### Type material.

***Holotype*** ♂ GP (NHRS): // NHRS-JLKB | 000066350 // Madagascar: Toamasina: Analanjirofo: | Masoala NP: lowalt. rainforest | MAD18-53: depression on forest floor | on path ~1.6 km NE of Andranobe camp |15.6713S, 49.9672E, 220 m, 18.II.2018 | Leg. J. Bergsten & T. Ranarilalatiana // Holotype | *Copelatus amphibius* sp. nov. | Det. Ranarilalatiana | & Bergsten, 2019 //

***Paratypes***: -7♂ GP, 6♀, 45 ex. (Alc.) (NHRS, DEUA & PBZT/MBC): // NHRS-JLKB | 000011230–1, 65651–2, 66016, 66347–9, 66351–5, 11232(Alc.) // Madagascar: Toamasina: Analanjirofo: | Masoala NP: lowalt. rainforest | MAD18-53: depression on forest floor | on path ~1.6 km NE of Andranobe camp |15.6713S, 49.9672E, 220 m, 18.II.2018 | Leg. J. Bergsten & T. Ranarilalatiana // Paratype | *Copelatus amphibius* sp. nov. | Det. Ranarilalatiana | & Bergsten, 2019 //

-3♂ GP, 5♀, 42 ex. (Alc.) (NHRS, DEUA & PBZT/MBC): // NHRS-JLKB | 000011234, 65649–50, 65788, 65790, 65795, 65435–6, 11233(Alc.) // Madagascar: Toamasina: Analanjirofo: | Nosy Mangabe, Masoala NP: MAD18-58 | rainfallpool with dead leaves nr path | after lighthouse, lowalt. rainforest | 15.5079S, 49.7641E, 195 m, 19.II.2018 | Leg. J. Bergsten & T. Ranarilalatiana // Paratype | *Copelatus amphibius* sp. nov. | Det. Ranarilalatiana | & Bergsten, 2019 //

-1♂ GP, 2♀ (NHRS): // NHRS-JLKB | 000065642–4 // Madagascar: Antsiranana: Sava: | Marojejy NP: midalt. rainforest: | small stream above camp II on | trail towards Taktajania, MAD18-23 | 14.4375S, 49.7612E, 860 m, 09.II.2018 | Leg. J. Bergsten & T. Ranarilalatiana // Paratype | *Copelatus amphibius* sp. nov. | Det. Ranarilalatiana | & Bergsten, 2019 //

#### Diagnosis.

A small species with medially infuscated testaceous elytra and oblong-oval body shape. Penis in lateral view with low ventral ”hump”, apical blade with acute apex and somewhat curved non-straight ventral margin, in ventral view apical blade is left-angled (Fig. 2A). Significantly smaller than described species from Madagascar with similar type of genitalia such as *C. owas* Régimbart, 1895 and *C. acamas* Guignot, 1955, and genitalia details also clearly different.

#### Description.

Body length: 4.6–5.4 mm (♀: 4.6–5 mm, ♂: 4.8–5.4 mm).

**Figure 1.**
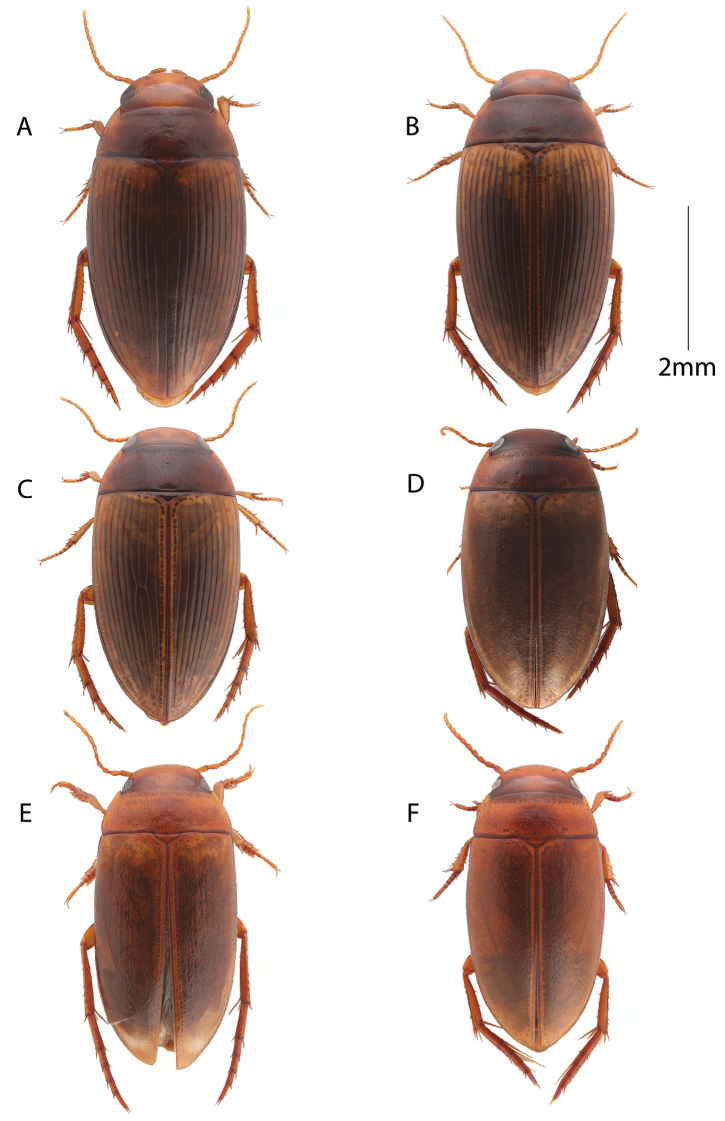
Habitus, dorsal view. **A***Copelatus amphibius* sp. nov. (female) **B***Copelatus zanatanensis* sp. nov. (female) **C***Copelatus betampona* sp. nov. (female) **D***Madaglymbus semifactus* sp. nov. (female) **E***Madaglymbus kelimaso* sp. nov. (male) **F***Madaglymbus menalamba* sp. nov. (female).

Body shape oval (Fig. 1A).

Head rufotestaceous with a rather weak v-shaped infuscation between eyes. Pronotum dark brown medially and testaceous laterally. Elytra testaceous brown, variably with darker infuscation medially especially along the striae (Fig. 1A). Basally, laterally and apically lighter testaceous. Appendages testaceous except metatarsus somewhat rufotestaceous.

Elytra with ten discal and one submarginal striae. Ninth striae avbreviated anteriorly. Submarginal striae present posteriorly only, starting at about middle. Posteriorly every second striae abbreviated (2^nd^, 4^th^, 6^th^, 8^th^, and 10^th^). Pronotum striolated laterally and basally. Lateral margin of pronotum with a narrow bead, not reaching anterior corner. Head, pronotum and elytra with same type of microreticulation and micropunctures.

Ventral side rufotestaceous except metacoxal plate infuscated brown. Abdominal sternites with vague testaceous spots laterally. Metacoxal plate with coarse strioles, abdominal sternites II–IV with finer strioles. Metacoxal lines anteriorly diverging and ending well before metaventral suture. Prosternal process lanceolate, short, and anterior metaventral process rather broad.

Male protibia modified, angled at base and expanding distally. Pro and mesotarsal segments I–III dilated and ventrally equipped with adhesive discs (constellation I:3 (row 1), 4 (row 2), II:4, III:4). Longer metatibial spur apically slightly more curved than in female.

**Figure 2.**
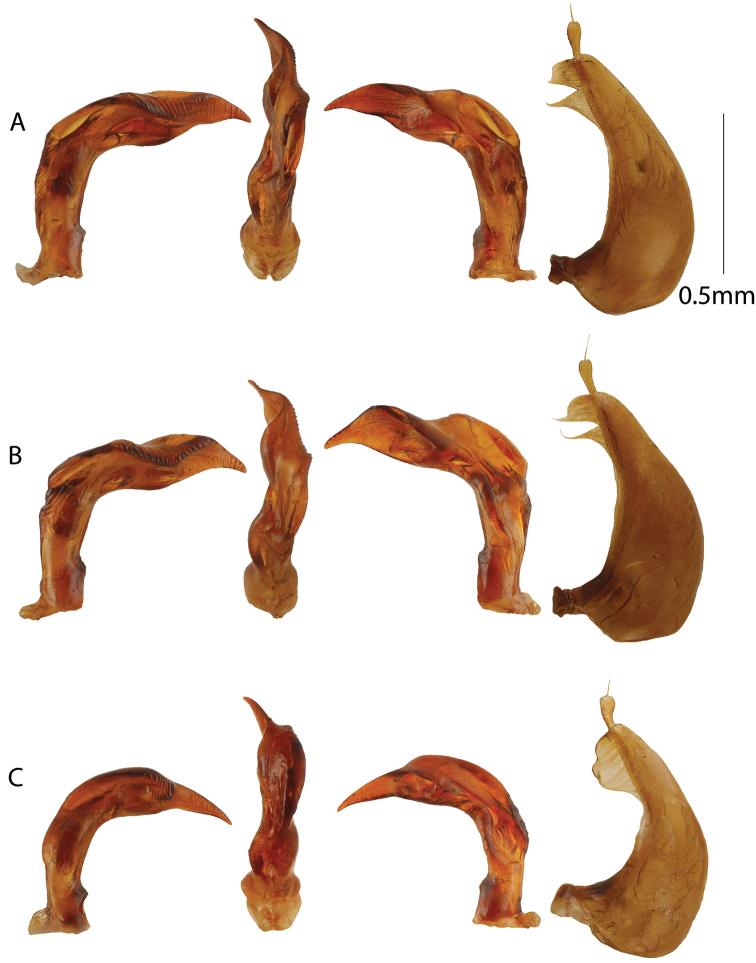
Male genitalia. From left to right aedeagus in right lateral, ventral, left lateral views, and left paramere. **A***Copelatus amphibius* sp. nov. **B***Copelatus zanatanensis* sp. nov. **C***Copelatus betampona* sp. nov.

Male genitalia as in Figure 2A. Penis curved and robust in lateral view with a comparatively low ventral hump and sinuate before the apical blade. Apical blade left-turned in ventral view. Right lateral side with strong rugosity or transverse ridges apically. Parameres as in Figure 2A.

Dorsal structures of females not significantly different from male, but body size on average smaller.

#### Distribution.

The northeastern humid forest from Marojejy NP to Masoala NP including the island of Nosy Mangabe (Fig. 4A).

#### Ecology and conservation.

*C. amphibius* sp. nov. was found in primary humid forests in dry shallow forest floor depression with dead leaves and soil at Masoala and in a rainwater-filled pool on Nosy Mangabe (Fig. 8). Both localities were at low altitudes but we also collected the species from Marojejy NP, in residual pools of a small stream at mid-altitude (860 m). Marojejy NP is unique in having continuous humid forest stretching from lowaltitude to the alpine zone and hence it is not surprising that the species can reach into the lower mid-altitude zone here. Both Marojejy and Masoala are since 2007 part of the UNESCO World Heritage Site Rainforests of the Atsinanana. Unfortunately, since 2010 Atsinanana is also on the list of World Heritage in Danger following a surge in illegal logging and hunting threatening its outstanding biodiversity values.

#### Etymology.

The Latin adjective amphibius comes from the ancient Greek word “amphibios” and means capable of living both in water and on land.

### Copelatus zanatanensis sp. nov.

Taxon classificationAnimaliaColeopteraDytiscidae

da1873fb-5659-5da8-866f-ac87b5e3a425

http://zoobank.org/4170CE38-15A3-4432-82EA-DF62700D935A

Figs 1B , 2B

#### Type locality.

Masoala National Park [15.6703S; 49.9715E] (Madagascar, Analanjirofo region, Maroantsetra)

#### Type material.

***Holotype*** ♂ GP (NHRS): NHRS-JLKB | 000011229 // Madagascar: Toamasina: Analanjirofo: | Masoala NP: lowalt. rainforest | MAD18-45: small muddy depression | on path ~2 km NE. of Andranobe camp | 15.6703S, 49.9715E, 360 m, 16.II.2018 | Leg. J. Bergsten & T. Ranarilalatiana // Holotype | *Copelatus zanatanensis* sp. nov. | Det. Ranarilalatiana | & Bergsten, 2019 //

***Paratypes***: -4♂ GP, 3♀, 17 ex. (Alc.) (NHRS, DEUA & PBZT/MBC): // NHRS-JLKB | 000065653, 66019, 66333–7, 11239(Alc.) // Madagascar: Toamasina: Analanjirofo: | Masoala NP: lowalt. rainforest | MAD18-43: dried out rainwater pool | on path ~3.5 km E. of Andranobe camp| 15.6681S, 49.9835E, 630 m, 16.II.2018 | Leg. J. Bergsten & T. Ranarilalatiana // Paratype | *Copelatus zanatanensis* sp. nov. | Det. Ranarilalatiana | & Bergsten, 2019 //

-5♂ GP, 5♀, 13 ex. (Alc.) (NHRS, DEUA & PBZT/MBC): // NHRS-JLKB | 000011237, 65654, 66017, 66326–32, 11238 (Alc.) // Madagascar: Toamasina: Analanjirofo: | Masoala NP: lowalt. rainforest | MAD18-44: pristine foreststream | 3 h walk (4 km) E of Andranobe camp | 15.6735S, 49.9886E, 500 m, 16.II.2018 | Leg. J. Bergsten & T. Ranarilalatiana // Paratype | *Copelatus zanatanensis* sp. nov. | Det. Ranarilalatiana | & Bergsten, 2019 //

-4♂ GP, 6♀, 6 ex. (Alc.) (NHRS, DEUA & PBZT/MBC): // NHRS-JLKB |000065655, 65787, 66018, 66302–6, 65437–8, 11228 (Alc.) // Madagascar: Toamasina: Analanjirofo: | Masoala NP: lowalt. rainforest | MAD18-45: small muddy depression | on path ~2 km NE. of Andranobe camp | 15.6703S, 49.9715E, 360 m, 16.II.2018 | Leg. J. Bergsten & T. Ranarilalatiana // Paratype | *Copelatus zanatanensis* sp. nov. | Det. Ranarilalatiana | & Bergsten, 2019 //

-6♂ GP, 4♀, 97 ex. (Alc.) (NHRS, DEUA & PBZT/MBC): // NHRS-JLKB | 000011235, 65656, 66325, 66362–8, 11236 (Alc.) // Madagascar: Toamasina: Analanjirofo: | Masoala NP: lowalt. rainforest | MAD18-49: small muddy depression | on path ~2 km NE. of Andranobe camp | 15.6703S, 49.9715E, 360 m, 18.II.2018 | Leg. J. Bergsten & T. Ranarilalatiana // Paratype | *Copelatus zanatanensis* sp. nov. | Det. Ranarilalatiana | & Bergsten, 2019 //

#### Diagnosis.

Habitus very similar to *C. amphibius* sp. nov. but penis diagnostic with a more distincly offset and higher ventral hump in lateral view and apical blade with a stright to almost concave ventral margin and spine-like acuminate apex (Fig. 2B).

#### Description.

Very similar in all respects to *C. amphibius* sp. nov. and only differences noted below.

Body length: 4.4–5 mm (♀: 4.4–4.8 mm, ♂: 4.7–5 mm).

On average slightly smaller and elytra less infuscated and therefore appearing more unicolorous lighter testaceous, but variation overlap between the species both in infuscation and body size (Fig. 1B).

Ventral side slightly lighter testaceous and therefore infuscation on metacoxal plate more contrasting.

Male genitalia as in Figure 2B. Penis diagnostic in lateral view with a straight to concave ventral margin of apical blade and an acuminate spine-like apex. *Copelatus amphibius* sp. nov. has a weakly convex ventral margin of the apical blade and apex pointed but not spine-like acuminate. Also, the ventral medial hump higher and more distinctly offset in *C. zanatanensis* sp. nov.

#### Distribution.

Only known from Masoala National Park, northeastern Madagascar (Fig. 4B).

#### Ecology and conservation.

*Copelatus zanatanensis* sp. nov. was found in forest floor depressions with leaf litter in pristine humid lowland (360–630 m alt.) forests in Masoala NP (Figs 6, 7). Most specimens were found in dry depressions at two different localities; however, the third place was in proximity of a stream, but still in a terrestrial microhabitat. Masoala National Park covers 230 000 ha of originally primary lowland humid forest, but deforestation rates surged following the political instability of 2009 (Allnutt et al. 2013).

#### Etymology.

Latinisation of the Malagasy word “zana-tany” litterally translated to “child of the land”, with the meaning to be native of a country. The new species is endemic and a native of Madagascar.

### Copelatus betampona sp. nov.

Taxon classificationAnimaliaColeopteraDytiscidae

5b3fccaa-42c5-5d35-839e-e74704ac5b7e

http://zoobank.org/35880EF1-1719-48F5-B8D2-D71BBC4B4D17

Figs 1C , 2C

#### Type locality.

Betampona Réserve Naturelle Intégrale (RNI) [17.9160S, 49.1999E] (Madagascar, Atsinanana region, Toamasina II)

#### Type material.

***Holotype*** ♂ GP (NHRS): // NHRS-JLKB | 000065440 // Madagascar: Toamasina: Atsinanana | Betampona RNI: lowalt rainforest | Path PPR, ca 100 m in from path PP | Dried out forest floor depression | MAD18-66: 24.II.2018 | 17.9160S, 49.1999E, 520 m | Leg. J. Bergsten & T. Ranarilalatiana // Holotype | *Copelatus betampona* sp. nov. | Det. Ranarilalatiana | & Bergsten, 2019 //

***Paratypes***: -6♂ GP, 8♀, 28 ex. (Alc.) (NHRS, DEUA & PBZT/MBC): // NHRS-JLKB | 000011227, 65659, 65786, 66015, 66338–46, 65439, 11226 (Alc.) // Madagascar: Toamasina: Atsinanana | Betampona RNI: lowalt rainforest | Path PPR, ca 100 m in from path PP | Dried out forest floor depression | MAD18-66: 24.II.2018 | 17.9160S, 49.1999E, 520 m | Leg. J. Bergsten & T. Ranarilalatiana // Paratype | *Copelatus betampona* sp. nov. | Det. Ranarilalatiana | & Bergsten, 2019 //

-3♂ GP, 24 ex. (Alc.) (NHRS, DEUA & PBZT/MBC): // NHRS-JLKB | 000010812, 11222, 65657, 11223 (Alc.) // Madagascar: Toamasina II: Betampona | RNI: MAD17-01: Vohimarangitra: | S-17.91604; E49.19986; 525 m: Dried | up forestpools in preaseape track: | 01/03/2017; Leg. T. Ranarilalatiana // Paratype | *Copelatus betampona* sp. nov. | Det. Ranarilalatiana | & Bergsten, 2019 //

-3♂ GP, 3♀ (NHRS): // NHRS-JLKB | 000011224–5, 65658, 66294–6 // Madagascar: Toamasina II: Betampona | RNI: MAD17-04: NW of park entrance: | S-17.93059; E49.20261; 321 m: Dried | up pools in Patsitsatra stream: | 03/03/2017; Leg. T. Ranarilalatiana // Paratype | *Copelatus betampona* sp. nov. | Det. Ranarilalatiana | & Bergsten, 2019 //

#### Diagnosis.

A slightly smaller species than preceding two and in fact the smallest of all known species of the *Copelatus erichsonii* group from Madagascar. Penis diagnostic in lateral view, lacking a sinuation between the ventral hump and the apical blade and with a long extended and narrow apical blade (Fig. 2C). *Copelatus gabonicus* Bilardo & Pederzani, 1978 and *Copelatus evanidus* Bilardo & Rocchi, 1995 (see figures in Bilardo and Rocchi 2015), both described from Gabon, have superficially similar type of genitalia, but differs in habitus and coloration as well as several genitalic details; the hump in lateral view is higher and more robust in *C. evadinus* and *C. gabonicus* have subapical transverse sulcation also in left lateral view.

#### Description.

Body length: 4.2–4.8 mm (♀: 4.2–4.6 mm, ♂: 4.6–4.8 mm).

Very similar in all respects to the two preceding species and only differences noted below.

Slightly smaller than both preceding species and somewhat less elongate (Fig. 1C).

The lightest testaceous species of all three. Elytra with very faint to no infuscation medially and infuscation between eyes on head essentially lacking (faint traces present).

Possibly more extensive striolation on pronotum, but individual variation likely to overlap between the species.

Metacoxal lines projecting anteriorly longer than in preceding two species but does not reach metaventral suture.

Male genitalia as in Figure 2C. Penis in lateral view diagnostic compared to preceding two species, with a ventral hump extending longer towards apex and without a sinuation between end of hump and beginning of apical blade. Apical blade lanceolate in shape with an evenly curved ventral margin. The large anterior portions of the asymetrically right-leaning hump also diagnostic in ventral view.

#### Distribution.

Only known from Betampona RNI, eastern lowland Madagascar (Fig. 4A).

#### Ecology and conservation.

*Copelatus betampona* sp. nov. was found in lowland humid forests (300–550 m alt.) in dry shallow depressions of the forest floor with dead leaves and soil (Fig. 5). Betampona RNI is managed through collaboration between Madagascar National Parks (MNP) and Madagascar Fauna and Flora Group (MFG). Betampona is one of the better-preserved low altitude parcels of rainforest on the eastern coast of Madagascar (Gehring et al. 2010; Rosa et al. 2012). It covers 2228 ha today, was until the late 1950s continuous with nearby forests but has since diminished and it is currently estimated that only around 50% of the area remains as primary forest (Britt 2002). Incursion by slash-and-burn agriculture likely represents the greatest threat to the biodiversity in the reserve. The fact that neither the *Madaglymbus* nor the *Copelatus* species found here were conspecific with those of lowland humid forests further north in Masoala indicates that Betampona, despite its small size, has a high conservation value for endemic eastern lowland fauna.

#### Etymology.

Named after the type locality and protected area where it was found, Betampona Réserve Naturelle Intégrale. The epithet is a noun in apposition.

### Madaglymbus

Taxon classificationAnimaliaColeopteraDytiscidae

Shaverdo & Balke, 2008

85fec7ac-4c18-5841-bca5-5746598950d4

#### Remark.

*Madaglymbus* was erected for the Madagascar species of *Aglymbus* Sharp, 1880 by Shaverdo et al. (2008). After Ranarilalatiana et al. (in press) transferred two *Copelatus* species to *Madaglymbus*, and including the three species described below, fifteen species and one subspecies are currently known from Madagascar and Comoros (see checklist below and Nilsson and Hájek 2019). We are constantly finding new species of this genus when collecting across Madagascar and it is premature to present a preliminary checklist including what is known but yet to be described. Revisionary work has been initiated with collegues and type material of all described species consulted directly or indirectly. The three species described below may not form a monophyletic group within the genus. They are described here in advance of a more complete revision of *Madaglymbus* as enigmatic representatives with the terrestrial habitats where they were collected in common.

##### Checklist

*M. alutaceus* (Régimbart, 1900) (Madagascar)

*M. apicalis* (Fairmaire, 1898) (Madagascar)

*M. elongatus* (H.J. Kolbe, 1883) (Madagascar)

*M. fairmairei* (Zimmermann, 1919) (Madagascar)

*M. formosulus* (Guignot, 1956) (Madagascar)

*M. johannis* (Wewalka, 1982) (Madagascar)

*M. kelimaso* Ranarilalatiana & Bergsten, 2019 sp. nov. (Madagascar)

*M. mathaei* (Wewalka, 1982) (Madagascar)

*M. menalamba* Ranarilalatiana & Bergsten, 2019 sp. nov. (Madagascar)

*M. milloti* (Guignot, 1959) (Comoros)

*M. ruthwildae* Shaverdo & Balke, 2008 (Madagascar)

*M. semifactus* Ranarilalatiana & Bergsten, 2019 sp. nov. (Madagascar)

*M. strigulifer* (Régimbart, 1903) (Madagascar)

M. strigulifer ssp. laevis (Guignot, 1955) (Madagascar)

*M. unguicularis* (Régimbart, 1903) (Madagascar)

*M. xanthogrammus* (Régimbart, 1900) (Madagascar)

### Madaglymbus semifactus sp. nov.

Taxon classificationAnimaliaColeopteraDytiscidae

881fbda0-1031-5f4f-aeed-0ba39be2c813

http://zoobank.org/0AF0971B-F955-4590-87A8-24BDCD7F3088

Figs 1D , 3A

#### Type locality.

Betampona Réserve Naturelle Intégrale (RNI) [17.9160S, 49.1999E] (Madagascar, Atsinanana region, Toamasina II)

#### Type material.

***Holotype*** ♂ GP (NHRS): // NHRS-JLKB | 000065445 // Madagascar: Toamasina II: Betampona | RNI: MAD17-01: Vohimarangitra: | S-17.91604; E49.19986; 525 m: Dried | up forestpools in preaseape track: | 01/03/2017; Leg. T. Ranarilalatiana // Holotype | *Madaglymbus semifactus* sp. nov. | Det. Ranarilalatiana | & Bergsten, 2019 //

***Paratypes***: -4♂ GP, 7♀ (NHRS, DEUA & PBZT/MBC): // NHRS-JLKB | 000066233, 66235–41, 66297–9 // Madagascar: Toamasina: Atsinanana | Betampona RNI: lowalt rainforest | Path PPR, ca 100 m in from path PP | Dried out forest floor depression | MAD18-66: 24.II.2018 | 17.9160S, 49.1999E, 520 m | Leg. J. Bergsten & T. Ranarilalatiana // Paratype | *Madaglymbus semifactus* sp. nov. | Det. Ranarilalatiana | & Bergsten, 2019 //

-2♀, 1♂ GP, 4♀ (Alc.) (NHRS): // NHRS-JLKB | 000065446–7, 65456, 65448 (Alc.) // Madagascar: Toamasina II: Betampona | RNI: MAD17-01: Vohimarangitra: | S-17.91604; E49.19986; 525 m: Dried | up forestpools in preaseape track: | 01/03/2017; Leg. T. Ranarilalatiana // Paratype | *Madaglymbus semifactus* sp. nov. | Det. Ranarilalatiana | & Bergsten, 2019 //

-2♀, 4♀ (Alc.) (NHRS): // NHRS-JLKB | 000065442–3, 65444 (Alc.) // Madagascar: Toamasina II: Betampona | RNI: MAD17-04: NW of park entrance: | S-17.93059; E49.20261; 321 m: Dried | up pools in Patsitsatra stream: | 03/03/2017; Leg. T. Ranarilalatiana // Paratype | *Madaglymbus semifactus* sp. nov. | Det. Ranarilalatiana | & Bergsten, 2019 //

-1♂ GP (NHRS): // NHRS-JLKB | 000065441 // Madagascar: Toamasina II: Analalava | reserve: MAD17-09: N of nursery | plants: S-17.70532; E49.45702; 75 m: | forest pools: 08/03/2017; Leg. T. | Ranarilalatiana // Paratype | *Madaglymbus semifactus* sp. nov. | Det. Ranarilalatiana | & Bergsten, 2019 //

#### Diagnosis.

**Figure 3.**
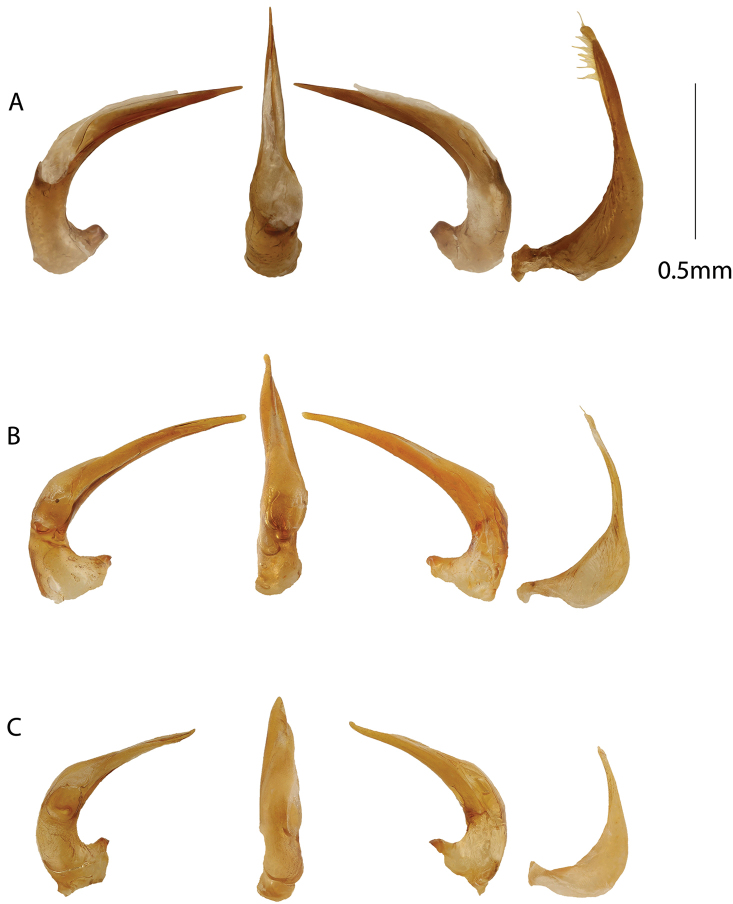
Male genitalia. From left to right aedeagus in right lateral, ventral, left lateral views, and left paramere. **A***Madaglymbus semifactus* sp. nov. **B***Madaglymbus kelimaso* sp. nov. **C***Madaglymbus menalamba* sp. nov.

A small *Madaglymbus* species, more oval than the two following species, with continuous outline between pronotum and elytra. Similar to *M. johannis*(Wewalka, 1982) but less elongate and easily distinguishable by the punctured elytra, punctures that are much finer than in *M. menalamba* sp. nov. Penis in ventral view short and straight (Fig. 3A), not right-angled towards apex as in *M. johannis*.

#### Description.

Body length: 3.7–4.2 mm (♀: 3.7–4.2 mm, ♂: 3.9–4.1 mm).

Body shape broadly oval with a continuous outline laterally between pronotum and elytra (Fig. 1D).

Head and pronotum rufous, infuscated inside eyes and vaguely medially on pronotum. Elytra infuscated medially but with testaceous sections basally, laterally and apically. Appendages testaceous except metatarsus rufotestaceous.

Elytra and pronotum covered with fine punctures, much finer than in *M. menalamba* sp. nov, but a distinguishing feature compared with the smooth elytra of *M. johannis*. Punctures on pronotum concentrated laterally with only micropunctures medially. Lateral marginal bead on pronotum thin and present only in posterior half. Head, pronotum and elytra with same type of microreticulation and micropunctures.

Ventral side rufotestaceous, metacoxal plate and abdominal sternites II–IV with few fine strioles laterally. Metacoxal lines absent. Anterior metaventral process broad.

Male pro and mesotarsal segments I–III dilated and ventrally equipped with adhesive discs (constellation I:3, 4, II:4, III:4). Anterodistal angle of protarsal segment IV with a modified stout seta.

Penis bilobed with ventral lobe extending to near, but stops before, apex of dorsal lobe. Penis straight, short and pointed with a thin apex in ventral view, straight and evenly tapering towards apex in lateral view (Fig. 3A). Compared with the longer and thinner penis of *M. johannis*, *M. semifactus* sp. nov. has a shorter and straighter penis, neither right-turned at apex in ventral view nor downturned in lateral view. Parameres with a rather long but broad apical extension (Fig. 3A).

Female similar to male.

#### Distribution.

**Figure 4.**
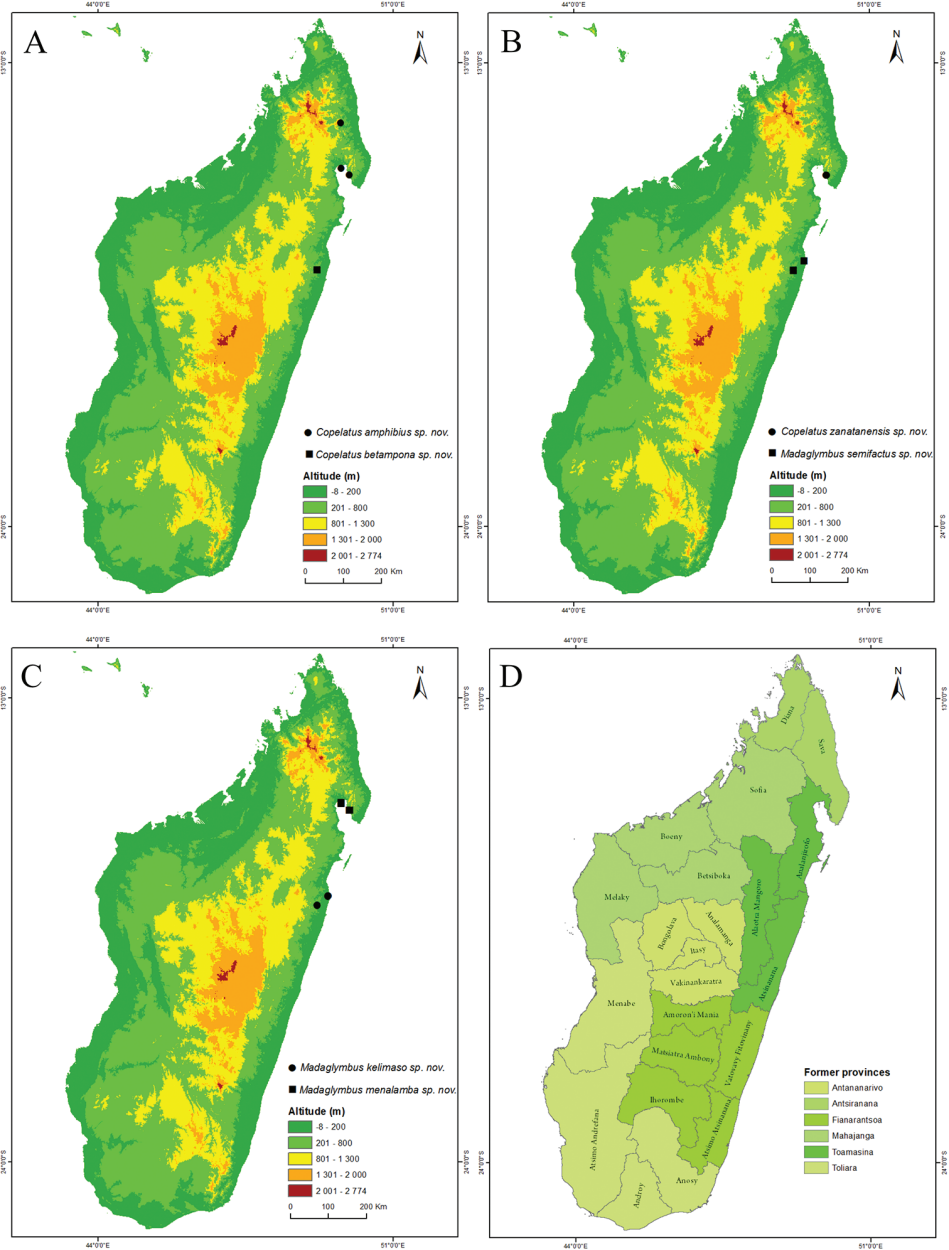
Maps of Madagascar with species distributions and administrative divisions. **A***Copelatus amphibius* sp. nov. (circle), *Copelatus betampona* sp. nov. (square) **B***Copelatus zanatanensis* sp. nov. (circle), *Madaglymbus semifactus* sp. nov. (square) **C***Madaglymbus kelimaso* sp. nov. (circle), *Madaglymbus menalamba* sp. nov. (square) **D** current 22 regions and six former provinces of Madagascar.

Known from Betampona RNI and at Analalava reserve, eastern lowland Madagascar (Fig. 4B).

#### Ecology and conservation.

**Figure 5.**
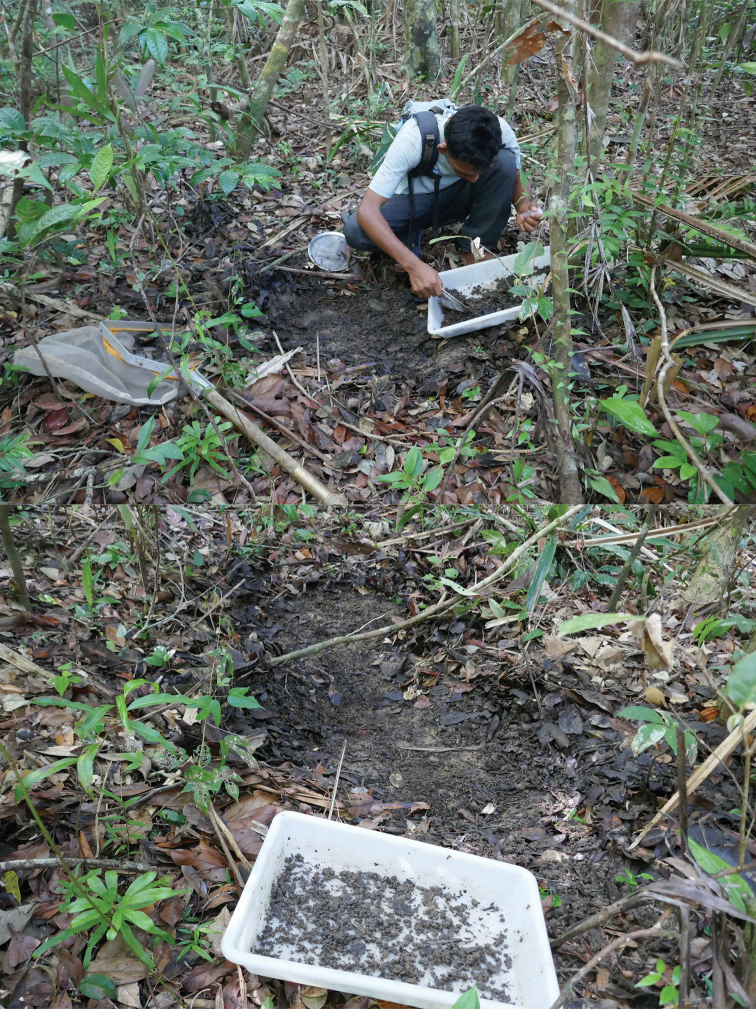
Habitat photo of locality MAD18-66, Betampona RNI. The three new species *Madaglymbus kelimaso* sp. nov., *Madaglymbus semifactus* sp. nov., and *Copelatus betampona* sp. nov. were found in this terrestrial habitat.

The species was found in Betampona RNI and collected under the same circumstances as *C. betampona* sp. nov. (Fig. 5). One specimen was collected from Analalava reserve in forest pools after a cyclone with heavy rain.

**Figure 6.**
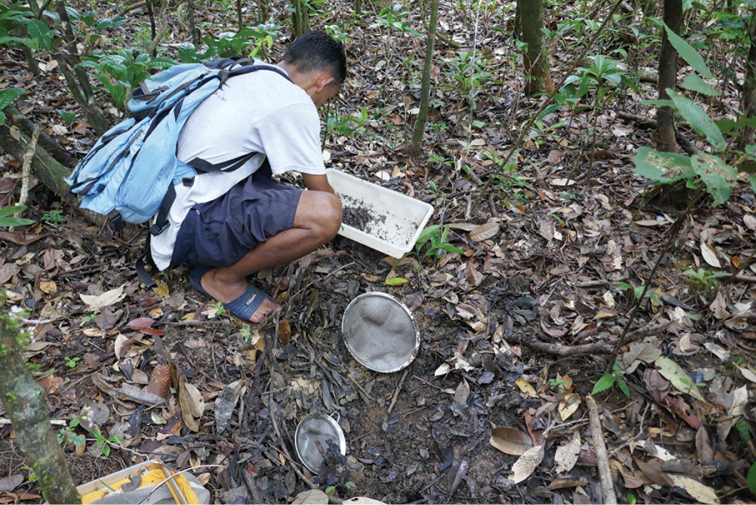
Habitat photo of locality MAD18-43, Masoala NP where *Copelatus zanatanensis* sp. nov. was found.

#### Etymology.

“Semifactus” means half-done or half-finished here referring to that this species is possibly less modified to spending time on land compared to next two *Madaglymbus* species.

### Madaglymbus kelimaso sp. nov.

Taxon classificationAnimaliaColeopteraDytiscidae

33ec148f-3ce6-5aaf-90ce-bb884bc02e4c

http://zoobank.org/9AE65691-3F6E-42F8-8BD3-91FDA4EB9953

Figs 1E , 3B

#### Type locality.

Analalava Reserve [17.70532S, 49.45702E] (Madagascar, Atsinanana region, Toamasina II)

#### Type material.

***Holotype*** ♂ GP (NHRS): // NHRS-JLKB | 000065449 // Madagascar: Toamasina II: Analalava | reserve: MAD17-06: Taniravo track: | S-17.70548; E49.45934; 52 m: forest | pools: 06/03/2017; Leg. T. | Ranarilalatiana // Holotype | *Madaglymbus kelimaso* sp. nov. | Det. Ranarilalatiana | & Bergsten, 2019 //

***Paratypes***: -2♀, 2♀ (Alc.) (NHRS): // NHRS-JLKB | 0000654450–1, 65452 (Alc.) // Madagascar: Toamasina II: Analalava | reserve: MAD17-06: Taniravo track: | S-17.70548; E49.45934; 52 m: forest | pools: 06/03/2017; Leg. T. | Ranarilalatiana // Paratype | *Madaglymbus kelimaso* sp. nov. | Det. Ranarilalatiana | & Bergsten, 2019 //

-3♀, 4♀ (Alc.) (NHRS): // NHRS-JLKB | 000065460–2, 65463 (Alc.) // Madagascar: Toamasina II: Analalava | reserve: MAD17-08: Lemur track: | S-17.70553; E49.45506; 64 m: forest | pools : 07/03/2017; Leg. T. | Ranarilalatiana // Paratype | *Madaglymbus kelimaso* sp. nov. | Det. Ranarilalatiana | & Bergsten, 2019 //

-1♂ GP, 4♀, 1♂ (Alc.), 8♀ (Alc.), (NHRS): // NHRS-JLKB | 000065464–8, 65469 (Alc.) // Madagascar: Toamasina II: Analalava | reserve: MAD17-09: N of nursery | plants: S-17.70532; E49.45702; 75 m: | forest pools: 08/03/2017; Leg. T. | Ranarilalatiana // Paratype | *Madaglymbus kelimaso* sp. nov. | Det. Ranarilalatiana | & Bergsten, 2019 //

-1♀, 1♀ (Alc.) (NHRS): // NHRS-JLKB | 000065454, 65455 (Alc.) // Madagascar: Toamasina II: Analalava | reserve: MAD17-10: N of nursery | plants: S-17.70812; E49.45171; 84 m: | forest pools beside Lemur track: | 08/03/2017; Leg. T. Ranarilalatiana // Paratype | *Madaglymbus kelimaso* sp. nov. | Det. Ranarilalatiana | & Bergsten, 2019 //

-1♂ GP, 1♀, 1♀ (Alc.) (NHRS): // NHRS-JLKB | 000065457–8, 65459 (Alc.) // Madagascar: Toamasina II: Betampona | RNI: MAD17-01: Vohimarangitra: | S-17.91604; E49.19986; 525 m: Dried | up forestpools in preaseape track: | 01/03/2017; Leg. T. Ranarilalatiana // Paratype | *Madaglymbus kelimaso* sp. nov. | Det. Ranarilalatiana | & Bergsten, 2019 //

-1♂ (Alc.) (NHRS): // NHRS-JLKB | 000065453 // Madagascar: Toamasina II: Betampona | RNI: MAD17-04: NW of park entrance: | S-17.93059; E49.20261; 321 m: Dried | up pools in Patsitsatra stream: | 03/03/2017; Leg. T. Ranarilalatiana // Paratype | *Madaglymbus kelimaso* sp. nov. | Det. Ranarilalatiana | & Bergsten, 2019 //

-1 ♂ GP (NHRS): // NHRS-JLKB | 000065789 // Madagascar: Toamasina: Atsinanana | Betampona RNI: lowalt rainforest | Path PPR, ca 100 m in from path PP | Dried out forest floor depression | MAD18-66: 24.II.2018 | 17.9160S, 49.1999E, 520 m | Leg. J. Bergsten & T. Ranarilalatiana // Paratype | *Madaglymbus kelimaso* sp. nov. | Det. Ranarilalatiana | & Bergsten, 2019 //

#### Diagnosis.

A small, elongate but rather robust *Madaglymbus* species with reddish coloration on head and pronotum and subrugose elytra with basal and apical testaceous spots (Fig. 1E). Penis evenly narrowing from base to apex in lateral view, non upturned at apex. Penis with bisinuate left side in ventral view and an apical knob is present in both ventral and lateral views (Fig. 3B). Parameres with a long and thin apical extension (Fig. 3B).

#### Description.

Body length: 3.9–4.8 mm (♀: 3.9–4.5 mm, ♂: 4.2–4.8 mm).

Body shape elongate, subparallell and rather convex. Lateral outline non-continuous between pronotum and elytra. Head broad with small eyes creating a wide interocular distance (Fig. 1E).

Pronotum and head rufotestaceous, infuscated inside eyes and slightly medially on pronotum. Elytra infuscated but with basal and apical testaceous spots. All appendages testaceous.

Elytra with longitudinal subugosity formed by shorter and longer strioles, sometimes connected to form longer continuous lines. Pronotum densely covered with large punctures and with a narrow lateral bead not reaching anterior corners. Head covered with finer punctation. Head, pronotum and elytra with same type of microreticulation and micropunctures.

Ventral side entirely testaceous, metacoxal lines absent but suggested ridge present in their place, metacoxal plate and abdominal sternites II–IV with fine strioles. Anterior metaventral process narrow.

Male pro and mesotarsal segments I–III broadly dilated and ventrally equipped with adhesive discs (constellation I:5 (row 1), 4 (row 2), II:4, III:4). Anterodistal angle of protarsal segment IV with a modified stout seta.

Bilobed penis with an apical knob visible in both lateral and ventral views, ventral lobe ending on right side well before apical knob of dorsal lobe. In lateral view apex not upturned (Fig. 3B). In ventral view left side bisinuate (Fig. 3B). Parameres with a long and thin apical extension (Fig. 3B).

Female with similar dorsal subrugosity as in male.

#### Distribution.

Known from Analalava reserve and Betampona RNI, eastern lowland Madagascar (Fig. 4C).

#### Ecology and conservation.

*Madaglymbus kelimaso* sp. nov. was found in lowland humid forests (50–550 m alt.). Most of the type specimens were found in Analalava reserve in forest pools with dead leaves, stagnant pools filled with water immediately after a cyclone with heavy rain (Fig. 11).

Analalava reserve is managed through collaboration between a local people NGO (Velonala) and Missouri Botanical Garden (MBG) since 2004. In 2015, it was designated as a new protected area. It covers 225 ha of typical littoral humid forest and represents one of few remaining forest fragment on the lowland central east coast of Madagascar. One specimen was collected in the same terrestrial habitat as *C. betampona* sp. nov. (Fig. 5).

#### Etymology.

The Malagasy word “kelimaso” means small eyes (keli = small, maso = eye), a characteristic of this species and seemingly an adaptation to spending significant amount of time out of water in the ground litter layer (three of five terrestrial dytiscid species are eyeless).

### Madaglymbus menalamba sp. nov.

Taxon classificationAnimaliaColeopteraDytiscidae

7b7f385f-4203-560d-8086-6c3a69266f89

http://zoobank.org/9D7D7816-63C3-476D-B272-9D326FFF95B5

Figs 1F , 3C , 12 , Suppl. material 1: Movie 1

#### Type locality.

Nosy Mangabe Special Reserve, part of Masoala National Park [15.4845S, 49.7627E] (Madagascar, Analanjirofo region, Maroantsetra)

#### Type material.

***Holotype*** ♂ GP (NHRS): // NHRS-JLKB | 000066360 // Madagascar:Toamasina:Analanjirofo: | Nosy Mangabe, Masoala NP: MAD18-63 | flat dry pansections of path btw camp | and Plage Hollandaise, lowalt. rainforest | 15.4845S, 49.7627E, 50 m, 20.II.2018 | Leg. J. Bergsten & T. Ranarilalatiana // Holotype | *Madaglymbus menalamba* sp. nov. | Det. Ranarilalatiana | & Bergsten, 2019 //

***Paratypes***: -4♂ GP, 2♀ (NHRS): // NHRS-JLKB | 000066010, 66307–11 // Madagascar: Toamasina: Analanjirofo: | Masoala NP: lowalt. rainforest | MAD18-45: small muddy depression | on path ~2 km NE. of Andranobe camp | 15.6703S, 49.9715E, 360 m, 16.II.2018 | Leg. J. Bergsten & T. Ranarilalatiana // Paratype | *Madaglymbus menalamba* sp. nov. | Det. Ranarilalatiana | & Bergsten, 2019 //

-4♂ GP, 10♀ (NHRS, DEUA & PBZT/MBC): // NHRS-JLKB | 000065792, 65794, 66009, 66314–24 // Madagascar: Toamasina: Analanjirofo: | Masoala NP: lowalt. rainforest | MAD18-49: small muddy depression | on path ~2 km NE. of Andranobe camp | 15.6703S, 49.9715E, 360 m, 18.II.2018 | Leg. J. Bergsten & T. Ranarilalatiana // Paratype | *Madaglymbus menalamba* sp. nov. | Det. Ranarilalatiana | & Bergsten, 2019 //

-1♂, 2♀ (NHRS): // NHRS-JLKB | 000066011–3 // Madagascar: Toamasina: Analanjirofo: | Masoala NP: lowalt. rainforest | MAD18-51: depression on forest floor on | path ~1.2 km NE of Andranobe camp, | 15.6735S, 49.9647E, 230 m, 18.II.2018 | Leg. J. Bergsten & T. Ranarilalatiana // Paratype | *Madaglymbus menalamba* sp. nov. | Det. Ranarilalatiana | & Bergsten, 2019 //

-3♂ GP, 2♀ (NHRS): // NHRS-JLKB | 000010814, 65791, 65793, 66012, 66293 // Madagascar: Toamasina: Analanjirofo: | Nosy Mangabe, Masoala NP: MAD18-57 | flat dry pansections of path after | lighthouse, lowalt. rainforest | 15.5078S, 49.7637E, 210 m, 19.II.2018 | Leg. J. Bergsten & T. Ranarilalatiana // Paratype | *Madaglymbus menalamba* sp. nov. | Det. Ranarilalatiana | & Bergsten, 2019 //

-1♂, 1♀ (NHRS): // NHRS-JLKB | 000066013, 66292 //Madagascar: Toamasina: Analanjirofo: | Nosy Mangabe, Masoala NP: MAD18-58 | rainfallpool with dead leaves nr path | after lighthouse, lowalt. rainforest | 15.5079S, 49.7641E, 195 m, 19.II.2018 | Leg. J. Bergsten & T. Ranarilalatiana // Paratype | *Madaglymbus menalamba* sp. nov. | Det. Ranarilalatiana | & Bergsten, 2019 //

-3♂, 2♀, 14 ex. (6♂, 8♀) (Alc.) (NHRS, DEUA & PBZT/MBC): // NHRS-JLKB | 000066356–9, 66361, 66014(Alc.) // Madagascar: Toamasina: Analanjirofo: | Nosy Mangabe, Masoala NP: MAD18-63 | flat dry pansections of path btw camp | and Plage Hollandaise, lowalt. rainforest | 15.4845S, 49.7627E, 50 m, 20.II.2018 | Leg. J. Bergsten & T. Ranarilalatiana // Paratype | *Madaglymbus menalamba* sp. nov. | Det. Ranarilalatiana | & Bergsten, 2019 //

#### Diagnosis.

A small and robust reddish *Madaglymbus* species similar to *M. kelimaso*, but less elongate and elytra concolorous with pronotum and covered with large punctures instead of subrugosity (Fig. 1F). Penis short and robust in ventral view with upturned apex in lateral view (Fig. 3C).

#### Description.

Body length: 3.7–4.5 mm (♀: 3.7–4.3 mm, ♂: 3.8–4.5 mm).

Body shape subparallell, robust and rather convex anteriorly. A broader body shape compared with *M. kelimaso* sp. nov. Lateral outline non-continuous between pronotum and elytra. Head broad with small eyes creating a wide interocular distance (Fig. 1F).

Body with a rather uniform reddish coloration, only head partly infuscated between eyes. All appendages testaceous except metatarsus rufotestaceous.

Elytra and pronotum densely covered with large punctures, puncturation reduced posteriorly and towards lateral margins of elytra. Pronotum with a narrow lateral bead not reaching anterior corners. Head covered with finer punctation. Head, pronotum and elytra with same type of microreticulation and micropunctures.

Ventral side entirely testaceous, metacoxal lines absent, suggestion of ridge in their place less distinct compared with *M. kelimaso* sp. nov., metacoxal plate and abdominal sternites II–IV with fine strioles. Anterior metaventral process broader than in *M. kelimaso* sp. nov.

Male pro and mesotarsal segments I–III dilated and ventrally equipped with adhesive discs (constellation I:3, 4, II:4, III:4). Segments less dilated than in *M. kelimaso* and first row with fewer discs. Anterodistal angle of protarsal segment IV with a modified stout seta.

Bilobed penis short and robust with rather blunt apex in ventral view. Ventral lobe twisted around right side of dorsal lobe to a position dorsal of it at apex (Fig. 3C). Apex in lateral view upturned. Parameres with apical extension not as long and thin as in *M. kelimaso* sp. nov. (Fig. 3C).

Female with similar dorsal puncturation as in male.

#### Distribution.

Masoala NP including the island of Nosy Mangabe (Fig. 4C).

#### Ecology and conservation.

**Figure 7.**
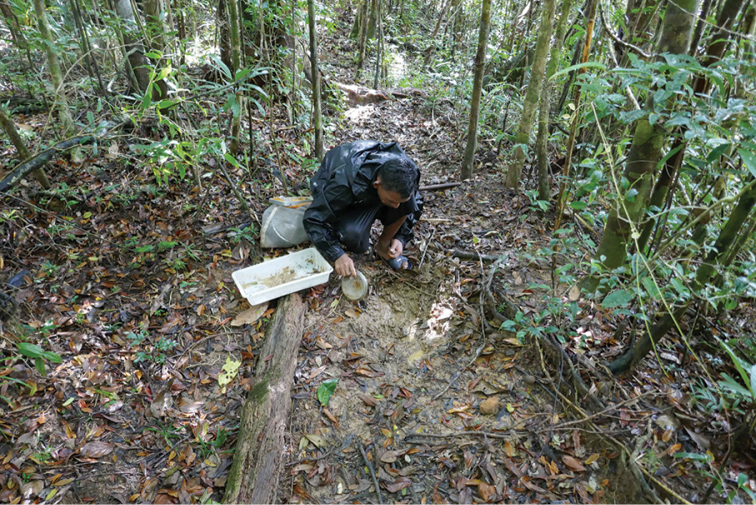
Habitat photos of locality MAD18-45, Masoala NP. The two new species *Copelatus zanatanensis* sp. nov. and *Madaglymbus menalamba* sp. nov. were found in this habitat.

**Figure 8.**
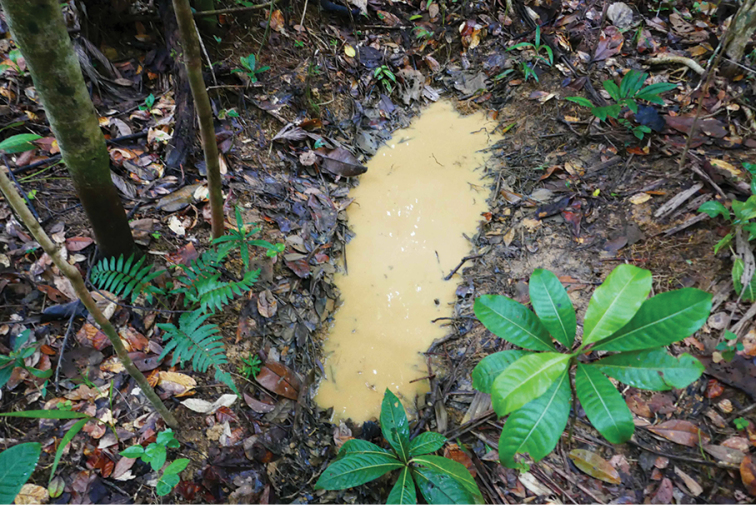
Habitat photo of locality MAD18-58, Nosy Mangabe, where the new species *Copelatus amphibius* sp. nov. and *Madaglymbus menalamba* sp. nov. were found. Of five different localities recorded for *M. menalamba* sp. nov. this was the only one where the species was found in water.

**Figure 9.**
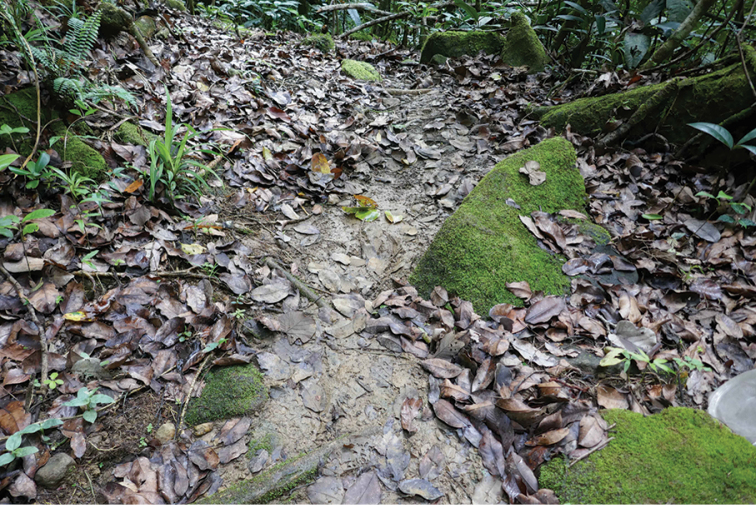
Habitat photo of locality MAD18-63, Nosy Mangabe, one of several similar localities where *Madaglymbus menalamba* sp. nov. was found.

**Figure 10.**
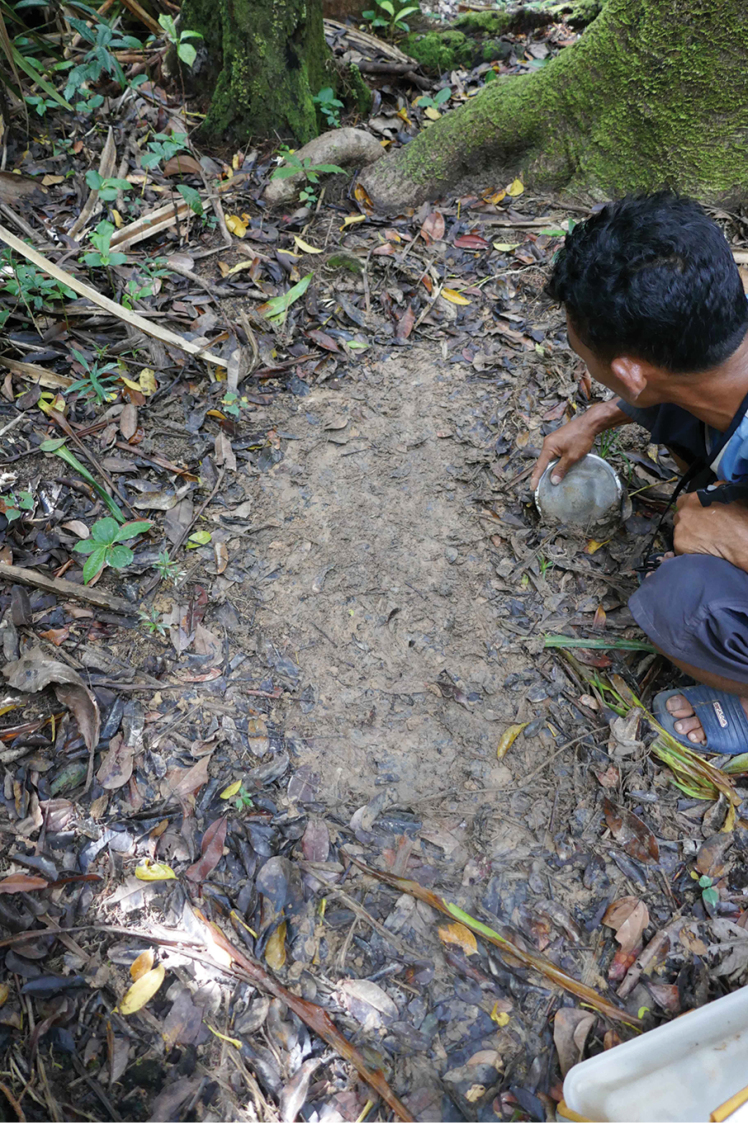
Habitat photo of locality MAD18-57, Nosy Mangabe, near where *Madaglymbus menalamba* sp. nov. was photographed and video-recorded.

The species was found in humid forests at low-altitude between (50–360 m) in dry shallow forest floor depression with dead leaves and soil. In one out of five localities it was collected from a rainwater-filled pit full of dead leaves, the other four places from dry forest floor depressions (Figs 7–10). Although less pristine and with clear signs of former human settlements, an equal number of specimens were found on Nosy Mangabe Island as compared with Masoala NP proper near Andranobe.

**Figure 11.**
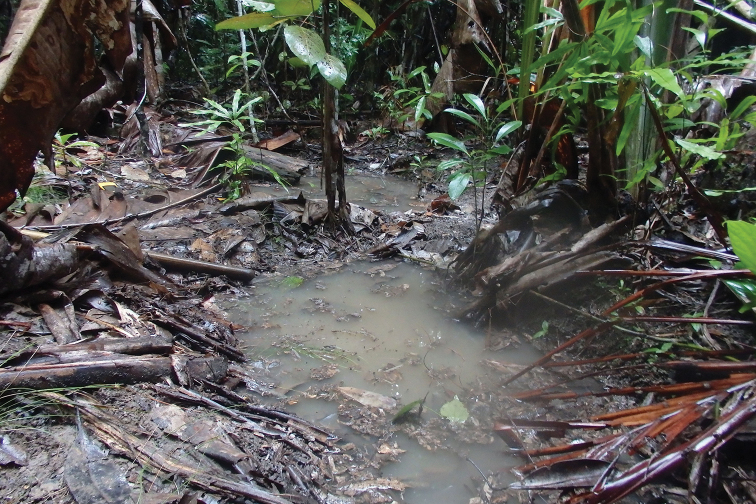
Habitat photo of locality MAD17-08, Analalava reserve, where the new species *Madaglymbus kelimaso* sp. nov. was found. These depressions were water-filled at time of visit 2017, immediately following a cyclone with heavy rain.

#### Etymology.

**Figure 12.**
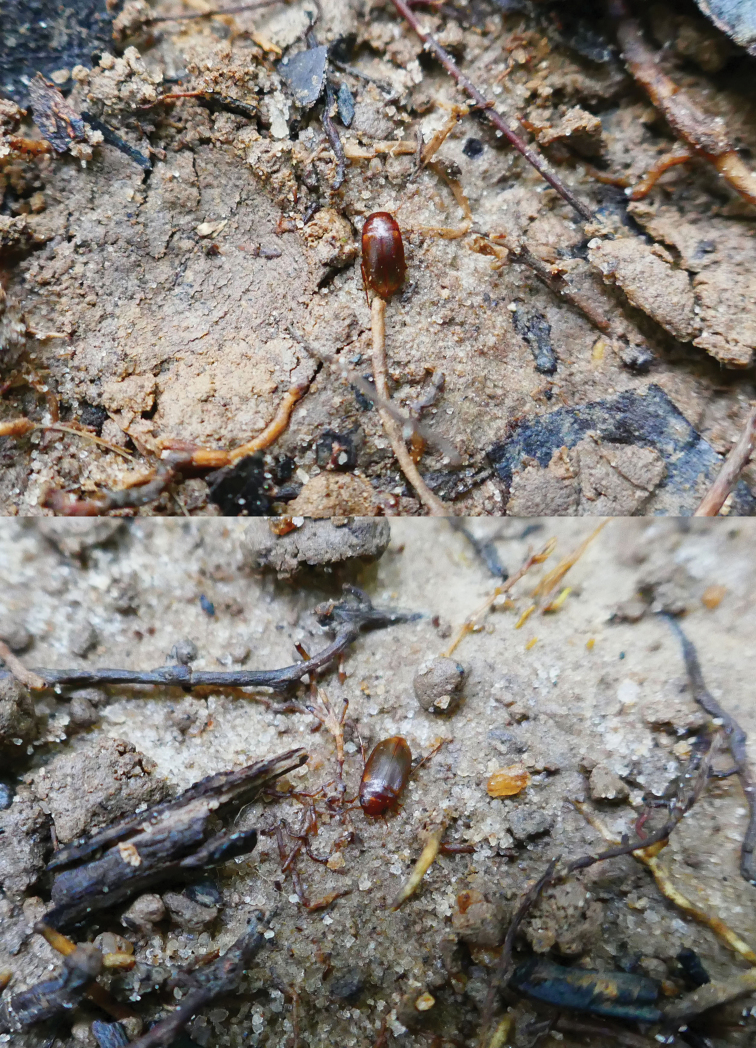
*Madaglymbus menalamba* sp. nov. photographed in the field on Nosy Mangabe (MAD18-57). Note the very distinctly reddish colour, not known from any previously described *Madaglymbus* species. See also Suppl. material 1: Movie 1 for recordings of the terrestrial locomotion (running) and behaviour of the species.

Menalamba in Malagasy means red clothes and the word is associated with the revolt and anti-colonianism movement in Madagascar’s history of independence. Here it refers to the characteristic reddish coloration of the species (Fig. 12, Suppl. material 1: Movie 1).

#### Notes.

At one locality on Nosy Mangabe (MAD18-57) we videorecorded the terrestrial locomotion and behaviour of this species in the field (Suppl. material 1: Movie 1). It was clearly apt at running and its immediate behaviour following exposure from, e.g. lifting up a dead leaf under which it was hiding, was to run and seek shelter again. This was repeated many times making it difficult to get photos of the species. The *Copelatus* species had a greater tendency to jump when exposed among the litter, but *M. menalamba* sp. nov. stayed put initially and then moved by running.

## Discussion

Copelatinae constitutes the second largest subfamily of diving beetles, with 759 species distributed in eight genera (Nilsson and Hájek 2019). The subfamily is represented by two genera on Madagascar, *Copelatus* Erichson, 1832 and *Madaglymbus* Shaverdo & Balke, 2008, and they are both diverse and widespread all over the island. The Malagasy Copelatinae was considered restricted to aquatic habitats and have likely never before been actively searched for in dry forest floor depressions. The discovery of a specialised fauna of *Copelatus* and *Madaglymbus* in this habitat was surprising. Although the inhabiting species should not be labelled terrestrial or even semi-terrestrial they have clearly adapted to withstanding periods of living, perhaps with reduced activity, terrestrial life in between heavy rains. Natatorial setae on legs are present and equivalent of those present in congeners collected aquatically why these species cannot be compared with the few terrestrial Hydroporinae taxa known with absent or much reduced natatorial setae on legs (Watts 1982; Brancucci 1979, 1985; Balke and Hendrich 1996). Rather they seem to have been able to occupy the for diving beetles unusual niche of the forest floor in a climatic zone with high rainfall regime. We hypothesise that this behavioural adaptation is restricted to humid forests with high annual precipitation. Andranobe in Masoala NP where *Copelatus amphibius* sp. nov., *C. zanatanensis* sp. nov., and *Madaglymbus menalamba* sp. nov. were found have the highest average (1993–1996) annual precipitation on Madagascar with 5900 mm, at least up to what had been measured up to 1996 (Jury 2003). A station in Sambava district measured an annual average of 3470 mm (2017–2018), which is probably an underestimate for Marojejy NP, although precipitation may vary with altitude and between slopes facing different directions (Goodman 2000). For Betampona and Analalava the closest station in Toamasina registered a yearly average of 2960 mm (2017–2018). In general, the northeast of Madagascar where all six species were found has the highest precipitation on the island. During the rainy season in the austral summer the northern third of Madagascar receives the greates rainfall, averaging 12 mm/day, which is in the upper 1% of rain intensity in the world (Jury 2003). In the dry season during austral winter, most of the island is dry, except the east coast where orographic uplifts of trade winds ensures year-around precipitation (Jury 2003). The northeast coast of the country therefore optimises both summer and winter precipitation levels. It is likely not a coincidence that this unusual specialist diving beetle fauna were discovered in this region of Madagascar. They are also very likely endemic to this region, or at the very least to humid forests on Madagascar. We collected the species during the rainy season 2017 and 2018. Future fieldwork during the dry-season in these regions would be very interesting to see if they still occupy the same certain-to-be-dry depressions also in this period or if they move to aquatic habitats. Larval development is most likely to take place during the rainy season, but it would also be interesting to know if it takes place in the same forest floor depressions or if more stable aquatic habitats are needed. The larval habitat of the few known truly terrestrial Hydroporinae species is thus far curiously unknown (Balke and Hendrich 2016). This conundrum aside, it is possible that inhabiting similar very ephemeral forest floor depressions was a stepping-stone, or transition step, towards becoming truly terrestrial.

The knowledge of Malagasy Copelatinae is still poor in general. Rocchi (1991) listed twenty-two species of *Copelatus* from Madagascar with twelve species from the *Copelatus erichsonii* group (Nilsson and Hájek 2019). Ranarilalatiana et al (in press) revised the non-*erichsonii* species groups and recognised thirteen species, out of which five were described as new. *Copelatus amphibius* sp. nov., *C. zanatanensis* sp. nov. and *C. betampona* sp. nov. falls in the *erichsonii*-group, based on the number of elytral striae (ten discal and one submarginal). They have in common not only the species group and specialised living but also the small body size, in fact smaller than all twelve previously described Malagasy species from the group. *Madaglymbus* is endemic to Madagascar and the Comoros Islands (Shaverdo et al. 2008; Miller and Bergsten 2016) and is now represented by fifteen described species and one subspecies (see checklist in Results section), although at least two times as many has been collected but are yet to be named and described. *Madaglymbus kelimaso* sp. nov., *M. menalamba* sp. nov. and *M. semifactus* sp. nov. are all three also in the smaller body size category within the genus. While the three *Copelatus* species do not portray any notable morphological attribute that may be an adaptation to terrestrial habits, the *Madaglymbus* species show unusual characteristics for the genus. The densely punctured (*M. menalamba* sp. nov. and *M. semifactus* sp. nov.) to subrugose (*M. kelimaso* sp. nov.) elytral surface is unique in this genus with otherwise typically shiny and smooth or more rarely striolate or aciculate elytra in other representatives. *Madaglymbus menalamba* sp. nov. and *M. kelimaso* sp. nov. additionally have an unusual reddish colour and have a more “caraboid” body shape with a non-continuous outline between pronotum and elytra. Whether any of these unusual characteristics are actual adaptations to terrestrial habits is not known, but the “caraboid” body shape may represent a higher degree of “terrestrialisation” in the latter two species compared to the other four. In the terrestrial Carabids, with few exceptions pronotum is narrower than elytra at posterior margin and this may enhance movability between pro- and mesothorax, and thereby manoeuvrability in the litter layer. The behaviour of rapid running and hiding when exposed, which was documented for *Madaglymbus menalamba* sp. nov. (Suppl. material 1: Movie 1), was very different to the jumping behaviour seen in the *Copelatus* species. This certainly seems to be a terrestrial adaptation - running requires alternate hind leg movement in contrast to the synchronous hind leg movement when swimming (and jumping). All new species herein described are endemic to Madagascar and could represent the “tip of the iceberg” as this habitat has just started to be explored. Similar forest-flooor specialist communities may also have evolved separately elsewhere in humid forests with high (year-around) precipitation. This is likely not a Madagascar-unique evolutionary trajectory, but to be in the upper one or few % rainfall intensity levels worldwide might be a necessary prerequisite.

## Supplementary Material

XML Treatment for Copelatus

XML Treatment for Copelatus amphibius

XML Treatment for Copelatus zanatanensis

XML Treatment for Copelatus betampona

XML Treatment for Madaglymbus

XML Treatment for Madaglymbus semifactus

XML Treatment for Madaglymbus kelimaso

XML Treatment for Madaglymbus menalamba

## Supplementary materials

Supplementary material 1 Movie 1 showing terrestrial locomotion of *Madaglymbus menalamba* sp. nov. Data type: video Explanation note: It was filmed at locality MAD18-57 on Nosy Mangabe Island, part of Masola NP, 18 February 2018. https://binary.pensoft.net/file/325120This dataset is made available under the Open Database License (http://opendatacommons.org/licenses/odbl/1.0/). The Open Database License (ODbL) is a license agreement intended to allow users to freely share, modify, and use this Dataset while maintaining this same freedom for others, provided that the original source and author(s) are credited.Tolotra Ranarilalatiana, Johannes Bergsten

## References

[B1] Allnutt TF Asner GP Golden CD Powell GVN (2013) Mapping recent deforestation and forest disturbance in northeastern Madagascar. Tropical Conservation Science 6: 1–15. 10.1177/194008291300600101

[B2] Balke M Dettner K Hendrich L (1997) *Agabus* (“Metronectes“) *aubei* Perris: Habitat, Morphological Adaptations, Systematics, Evolution, and Notes on the Phanaerofluicolous Fauna (Coleoptera: Dytiscidae). Aquatic Insects 19(2): 75–90. 10.1080/01650429709361640

[B3] Balke M Hendrich L (1996) A new species of the terrestrial water beetle genus Geodessus Brancucci (Coleoptera: Dytiscidae), sieved from leaf litter in southern India. Aquatic Insects 18: 91–99. 10.1080/01650429609361607

[B4] Balke M Hendrich L (2016) Dytiscidae. In: Beutel RG Leschen RAB (Eds) Handbook of Zoology.Coleoptera, beetles. Volume 1: Morphology and Systematics (Archostemata, Adephaga, Myxophaga, Polyphaga partim) (2^nd^ edn). DeGruyter, Berlin, 118–140.

[B5] Bilardo A Rocchi S (2015) A revision and synopsis of the African species of the genus Copelatus Erichson, 1832. The group erichsonii, subgroup atrosulcatus (ColeopteraDytiscidae). Memorie della Società Italiana di Scienze Naturali e del Museo Civico di Storia Naturale di Milano 40: 1–38.

[B6] Bilton DT (2014) Dispersal in Dytiscidae. In: Yee DA (Ed.) Ecology, Systematics, and the Natural History of Predaceous Diving Beetles (Coleoptera: Dytiscidae).Springer, Netherlands, 387–407. 10.1007/978-94-017-9109-0_9

[B7] Brancucci M (1979) *Geodessus besucheti* n. gen., n. sp. le premier dytiscide terrestre (Col., Dytiscidae, Bidessini). Entomologica Basiliensia 4: 213–218.

[B8] Brancucci M (1985) A review of the biology and structure of *Geodessus besucheti* Brancucci (Coleoptera, Dytiscidae). Proceedings of the Acedmy of Natural Sciences of Philadelphia 137: 29–32.

[B9] Brancucci M Hendrich L (2010) Dytiscidae: *Typhlodessus monteithi* Brancucci, 1985, redescription and notes on habitat and sampling circumstances (Coleoptera). In: Jäch MA, Balke M (Eds) Water Beetles of New Caledonia (Part 1), Monographs on Coleoptera 3: 163–170.

[B10] Britt A (2002) Observations on Two Dwarf Dypsis Species in Betampona, Eastern Madagascar. Palms 46: 125–129. http://www.palms.org/palmsjournal/2002/vol46n3p125-129.pdf

[B11] Gehring PS Ratsoavina FM Vences M (2010) Filling the gaps: amphibian and reptile records from lowland rainforests in eastern Madagascar. Salamandra 46: 214–234. http://www.salamandra-journal.com/index.php/home/contents/2010-vol-46/223-gehring-p-s-f-m-ratsoavina-m-vences/file

[B12] Gioria M (2014) Habitats. In: Yee DA (Ed.) Ecology, Systematics, and the Natural History of Predaceous Diving Beetles (Coleoptera: Dytiscidae).Springer, Netherlands, 307–362. 10.1007/978-94-017-9109-0_7

[B13] Goodman SM (2000) Chapter 1: Description of the Parc National de Marojejy, Madagascar, and the 1996 Biological Inventory of the Reserve. In: Goodman SM (Ed.) A floral and faunal inventory of the Parc National de Marojejy, Madagascar: with reference to elevational variation.Fieldiana Zoology, Chicago: Field Museum of Natural History 97: 1–18. 10.5962/bhl.title.3276

[B14] Goodman SM Benstead JP (2005) Updated estimates of biotic diversity and endemism for Madagascar. Oryx 39: 73–77. 10.1017/S0030605305000128

[B15] Jury MR (2003) The Climate of Madagascar. In: Goodman MA Benstead JP (Eds) The Natural History of Madagascar.The University of Chicago Press. Chicago, 75–87.

[B16] Jäch MA (1998) Annotated check list of aquatic and riparian/littoral beetle families of the world (Coleoptera). In: Jäch MA Ji L (Eds) Water Beetles of China vol.II. Wien: Zoologisch-Botanische Gesellschaft in Österreich and Wiener Coleopterologenverein, 25–42.

[B17] Jäch MA Balke M (2008) Global diversity of water beetles (Coleoptera) in freshwater. Hydrobiologia 595: 419–442. 10.1007/978-1-4020-8259-7_43

[B18] Máiz-Tomé L Sayer C Darwall W (2018) The status and distribution of Freshwater Biodiversity in Madagascar and the Indian Ocean Islands hotspot. IUCN Freshwater Biodiversity Unit, Global Species Programme. IUCN Cambridge, UK, 128 pp 10.2305/IUCN.CH.2018.RA.1.en

[B19] Miller KB Bergsten J (2016) Diving beetles of the World. Systematics and biology of the Dytiscidae. Johns Hopkins University Press, Baltimore, USA, 320 pp.

[B20] Myers N Mittermeier RA Mittermeier CG Da Fonseca GA Kent J (2000) Biodiversity hotspots for conservation priorities. Nature 403: 853–858. 10.1038/35002501 10706275

[B21] Nilsson AN Hájek J (2019) A world catalogue of the family Dytiscidae, or the diving beetles (Coleoptera, Adephaga). Version 1.I.2019, 307 pp. http://www.waterbeetles.eu/documents/W_CAT_Dytiscidae_2019.pdf

[B22] Ranarilalatiana T Raveloson Ravaomanarivo LH Bergsten J (in press) Taxonomic revision of the genus *Copelatus* of Madagascar (Coleoptera, Dytiscidae, Copelatinae): the non-erichsonii group species. ZooKeys. 10.3897/zookeys.869.33997 PMC6690892 31413659

[B23] Rocchi S (1991) Contributo alla conoscenza degli Haliplidae e dei Dytiscidae del Madagascar con descrizione di due nuove specie (Coleoptera). Frustula Entomologica (Nuova Serie) 14: 71–89.

[B24] Rosa GM Andreone F Crottini A Hauswaldt JS Noël J Rabibisoa NH Randriambahiniarime MO Rebelo R Raxworthy CJ (2012) The amphibians of the relict Betampona low-elevation rainforest, eastern Madagascar: An application of the integrative taxonomy approach to biodiversity assessments. Biodiversity and Conservation 21: 1531–1559. 10.1007/s10531-012-0262-x

[B25] Shaverdo HV Monaghan MT Lees DC Ranaivosolo R Balke M (2008) *Madaglymbus*, a new genus of Malagasy endemic diving beetles and description of a highly unusual species based on morphology and DNA sequence data (Dytiscidae: Copelatinae). Systematics and Biodiversity 6: 43–51. 10.1017/S1477200007002599

[B26] Toussaint EFA Hendrich L Escalona HE Porch N Balke M (2016) Evolutionary history of a secondary terrestrial Australian diving beetle (Coleoptera, Dytiscidae) reveals a lineage of high morphological and ecological plasticity. Systematic Entomology 41: 650–657. 10.1111/syen.12182

[B27] Watts CHS (1982) A blind terrestrial water beetle from Australia. Memoires of the Queensland Museum 20: 527–531.

